# Modeling Calcium Signaling in *S. cerevisiae* Highlights the Role and Regulation of the Calmodulin-Calcineurin Pathway in Response to Hypotonic Shock

**DOI:** 10.3389/fmolb.2022.856030

**Published:** 2022-05-18

**Authors:** Simone Spolaor, Mattia Rovetta, Marco S. Nobile, Paolo Cazzaniga, Renata Tisi, Daniela Besozzi

**Affiliations:** ^1^ Department of Informatics, Systems and Communication, University of Milano-Bicocca, Milan, Italy; ^2^ Department of Environmental Sciences, Informatics and Statistics, Ca’ Foscari University of Venice, Venice, Italy; ^3^ Bicocca Bioinformatics, Biostatistics and Bioimaging Centre—B4, Milan, Italy; ^4^ SYSBIO/ISBE.IT Centre of Systems Biology, Milan, Italy; ^5^ Department of Human and Social Sciences, University of Bergamo, Bergamo, Italy; ^6^ Department of Biotechnology and Biosciences, University of Milano-Bicocca, Milan, Italy

**Keywords:** calcium signaling, hypotonic shock response, mathematical model, Saccharomyces cerevisiae, calmodulin-calcineurin pathway

## Abstract

Calcium homeostasis and signaling processes in *Saccharomyces cerevisiae*, as well as in any eukaryotic organism, depend on various transporters and channels located on both the plasma and intracellular membranes. The activity of these proteins is regulated by a number of feedback mechanisms that act through the calmodulin-calcineurin pathway. When exposed to hypotonic shock (HTS), yeast cells respond with an increased cytosolic calcium transient, which seems to be conditioned by the opening of stretch-activated channels. To better understand the role of each channel and transporter involved in the generation and recovery of the calcium transient—and of their feedback regulations—we defined and analyzed a mathematical model of the calcium signaling response to HTS in yeast cells. The model was validated by comparing the simulation outcomes with calcium concentration variations before and during the HTS response, which were observed experimentally in both wild-type and mutant strains. Our results show that calcium normally enters the cell through the High Affinity Calcium influx System and mechanosensitive channels. The increase of the plasma membrane tension, caused by HTS, boosts the opening probability of mechanosensitive channels. This event causes a sudden calcium pulse that is rapidly dissipated by the activity of the vacuolar transporter Pmc1. According to model simulations, the role of another vacuolar transporter, Vcx1, is instead marginal, unless calcineurin is inhibited or removed. Our results also suggest that the mechanosensitive channels are subject to a calcium-dependent feedback inhibition, possibly involving calmodulin. Noteworthy, the model predictions are in accordance with literature results concerning some aspects of calcium homeostasis and signaling that were not specifically addressed within the model itself, suggesting that it actually depicts all the main cellular components and interactions that constitute the HTS calcium pathway, and thus can correctly reproduce the shaping of the calcium signature by calmodulin- and calcineurin-dependent complex regulations. The model predictions also allowed to provide an interpretation of different regulatory schemes involved in calcium handling in both wild-type and mutants yeast strains. The model could be easily extended to represent different calcium signals in other eukaryotic cells.

## 1 Introduction

Calcium ions (Ca^2+^) have many physiological functions and are ubiquitously used by prokaryotic and eukaryotic unicellular organisms, as well as by multicellular eukaryotes (Plattner and Verkhratsky, 2015b; Plattner and Verkhratsky, 2015a; Giorgi et al., 2018; Bagur and Hajnóczky, 2017). Ca^2+^ represents a universal intracellular messenger that modulates a plethora of processes, such as the control of cell proliferation, programmed cell death, neurotransmission, secretion, vesicular transport, cytoskeleton rearrangement, and transcription (Berridge et al., 2000). Ca^2+^ sequestration in different cellular compartments is the key to maintaining appropriate concentration gradients, which are required to generate the temporal and spatial patterns exploited by cells to encode signals about their own status or the extracellular environment (Berridge et al., 2003; Dupont et al., 2007; Purvis and Lahav, 2013). Specific Ca^2+^ signals are triggered only upon an intra- or extra-cellular stimulus; in the absence of such events, the cell must maintain properly low and non-signaling Ca^2+^ concentrations, in a narrow range of 50–200 nM in eukaryotic cells. This feature is implemented by an extensive and well conserved cellular toolkit comprised of Ca^2+^-sensing proteins, buffers, channels, pumps, and exchangers (Plattner and Verkhratsky, 2015b). Although Ca^2+^ signals could possibly be generated in all cell compartments, they are mainly studied in the cytosol, and are the result of the release from intracellular stores or the influx from the extracellular environment, or both. The disruption of Ca^2+^ homeostasis system can potentially lead to unwanted (in)activation of signaling cascades, and in turn cause cell defects or even cell death (Carafoli, 2004; Sammels et al., 2010).

To help unraveling the role of channels and transporters in maintaining Ca^2+^ homeostasis, in this work we provide a mathematical model of the hypotonic shock (HTS) response in budding yeast cells. HTS consists in a sudden variation of the osmotic pressure, due to a consistent dilution of the solution concentration to which the cell is exposed. Following a HTS, water flows into the cell, causing an increase in cell volume and turgor pressure that might induce the cell burst. To avoid cytolysis, yeasts have evolved mechanisms to sense and respond to HTS, by rapidly triggering at least three different mechanisms: 1) the cytosolic Ca^2+^ concentration is transiently increased; 2) the osmolyte glycerol is released to the medium in order to relieve osmotic pressure; 3) phospholypase C hydrolizes PI(4,5)P_2_ generating the major second messengers, diacylglycerol (DAG) and inositol-(3,4,5)-*tris*-phosphate (IP_3_). In mammalian cells, DAG is well-known as a protein kinase C activator, while IP_3_ is involved in triggering the release of Ca^2+^ from intracellular compartments such as endoplasmic reticulum or Golgi (Pinton et al., 1998; Berridge et al., 2000); it is not clear if this applies to yeast as well, but a role for inositol phosphate in calcium release was previously reported (Belde et al., 1993; Tisi et al., 2002, 2004).

The yeast *S. cerevisiae* has evolved a cell wall that, having less elasticity than the plasma membrane, prevents the cell from excessive expansion (Aguilar-Uscanga and Francois, 2003; Alsteens et al., 2008; Orlean, 2012). The adaptation of the cell wall to the environmental challenges is controlled by the cell wall integrity (CWI) signaling pathway, which shows a complex interrelationship with Ca^2+^ signaling (Hohmann, 2002; Levin, 2011).

The mathematical model presented in this work was defined by integrating well-established experimental evidences with plausible hypotheses on the functioning of HTS response. The main components of the model comprise: 1) the Ca^2+^ membrane transporters, namely, the High Affinity Calcium influx System (HACS) and mechanosensitive channels (MS); 2) the vacuolar transporters Pmc1 and Vcx1; 3) the Ca^2+^-binding messenger protein calmodulin (CaM), and the Ca^2+^-CaM-dependant phosphatase calcineurin (CaN). The model is formalised as a system of coupled Ordinary Differential Equations (ODEs), and it can be conceptually divided in two modules: a *biophysical module*, describing the physical properties of yeast cells (e.g., volume, turgor pressure, and membrane tension), and a *biochemical module*, describing the changes in the concentration of protein and molecules involved in Ca^2+^ signaling upon HTS, and including the feedback regulation via the calmodulin-calcineurin pathway.

The model simulations are in accordance with published experimental results, suggesting that the biophysical and the biochemical modules are able to explain the role and interplay among the essential components involved in the HTS response. Our analysis shows that calcium enters the resting cell through the HACS and the MS channels. However, upon HTS, the increase of the plasma membrane tension amplifies the opening probability of MS channels, thus causing a sudden Ca^2+^ pulse. The rapid recovery of the basal Ca^2+^ levels in the cytosol primarily involves Pmc1, differently than in other signalling processes where massive amounts of Ca^2+^ enter the cytoplasm requiring Vcx1 to engage in the recovery (Miseta et al., 1999b). According to our simulations, the involvement of Vcx1 is actually marginal, unless Vcx1 inhibition by calcineurin is removed. Our results also suggest that the MS channels are subject to a calcium-dependent feedback inhibition, possibly involving calmodulin, since it is not relieved by calcineurin removal. This suggests that the very sharp signature of HTS-induced calcium peak would be obtained by the rapid closure of the MS channels triggered by this feedback loop.

The paper is organized as follows: Section 2 provides a detailed description of the cellular components and processes that were taken into account in the HTS response model; in Section 3 we explain how the two modules of the model were formalised and simulated; Section 4 provides a detailed description of the modules and their parameters; Section 5 shows the results we obtained by comparing experimental data with our model simulations; finally, we discuss these results in Section 6 and draw final conclusions in Section 7.

## 2 Biological Background

**FIGURE 1:**
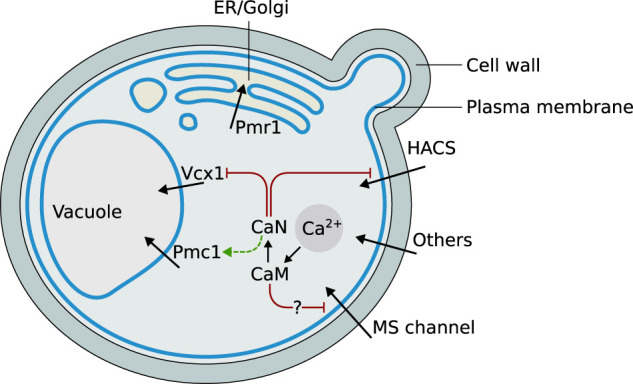
Graphical schematization of the role and localization of the Ca^2+^ signaling components included in the model, and their mutual regulations. Abbreviations: ER = endoplasmic reticulum, HACS = High Affinity Calcium influx System, CaN = calcineurin, CaM = calmodulin, MS = mechanosensitive.

The main cellular components appearing in the mathematical model are represented in Figure 1. Their biological function and mutual regulation in controlling Ca^2+^ homeostasis and signaling, especially upon HTS, are described in the following sections.

### 2.1 Calcium Transport and Homeostasis

#### 2.1.1 Membrane Transporters

The High Affinity Calcium influx System (HACS) is composed of Cch1 and Mid1. Cch1 is a homolog of the pore-forming *α*1 subunit of mammalian voltage-gated Ca^2+^ channels (Fischer et al., 1997), while Mid1 is a stretch-activated Ca^2+^-permeable nonselective cation channel whose secondary structure is similar to the non-pore-forming *α*2*δ* subunit that associates with the mammalian *α*1 (Iida et al., 1994). Cch1 and Mid1 cooperate in many yeast processes, such as mating pheromone-induced Ca^2+^ uptake (Paidhungat and Garrett, 1997), store-operated Ca^2+^ entry (Locke et al., 2000), ER stress-induced Ca^2+^ uptake (Bonilla et al., 2002), and hyperosmotic stress-induced increase of cytosolic Ca^2+^ (Matsumoto et al., 2002). Cch1 seems to respond to membrane depolarization, as the HACS is dependent on the presence of Kch1 and Kch2, two putative potassium transporters that mediate K^+^ influx and, likely, plasma membrane depolarization in pheromone-induced conditions (Stefan C. P. et al., 2013), as well as upon ER stress (Stefan and Cunningham, 2013) or glucose addition (Ma et al., 2021). Some lines of evidence suggest that Mid1 and Cch1 can also function independently from each other (Kanzaki et al., 1999), in different compartments (Yoshimura et al., 2004) or upon different stimuli (Courchesne and Ozturk, 2003).

Besides other yet poorly characterized Ca^2+^ influx systems in the plasma membrane (Eilam and Othman, 1990; Muller et al., 2001; Muller et al., 2003; Groppi et al., 2011), the presence of another plasma membrane Ca^2+^ influx system was implied based on the fact that the hypotonic stress-induced [Ca^2+^]_cyt_ increase was not inhibited by removing all known transporters (Rigamonti et al., 2015). Its molecular identity is unknown but it is likely to include a Transient Receptor Potential (TRP)-like protein, Flc2, which provides the channel with a [Ca^2+^]_ext_-dependent inhibition. Flc2 is a member of the fungal spray family, which comprises TRP-like poly-cystic-kidney-disease (PKD)-related calcium channels (Tisi et al., 2016). TRP channels are well conserved and their regulation is polymodal. Almost all TRP channels appear to function as homo- or hetero-tetramers. TRP channels are regulated by a very large spectrum of chemical and physical stimuli such as phosphoinositides, Ca^2+^, cyclic nucleotides, temperature, voltage, osmotic stress, and membrane shearing. A single TRP channel can exhibit sensitivity to multiple types of stimuli and thus mediates integrated responses (Zheng, 2013).

Differently than in plant and animal cells, Ca^2+^ efflux proteins have not been detected on the plasma membrane of yeast cells. However, Ca^2+^ is presumably excluded from the cytoplasm by yet unknown Ca^2+^ transport mechanisms. Early experiments showed that the presence of potassium or sodium in the medium—and their consequent influx in the cells—induces efflux of Ca^2+^ (Eilam, 1982b). Analogous results were obtained with low external pH, suggesting the presence of a Ca^2+^/H^+^ antiport located on the plasma membrane (Eilam, 1982a; Hong et al., 2013).

#### 2.1.2 Vacuolar Transporters.

Ca^2+^ is massively stored in the vacuole by two active transporters: Pmc1, a high affinity low capacity Ca^2+^-ATPase (Cunningham K. and Fink G., 1994), and Vcx1, a low affinity high capacity Ca^2+^/H^+^ exchanger. Vcx1 has a major role in shaping the calcium signal, since its high capacity can rapidly attenuate a large burst of cytosolic Ca^2+^ concentration (Miseta et al., 1999b). The free Ca^2+^ concentration in the budding yeast vacuole is estimated to be of about 30 *μ*M, although larger amounts are stored as inorganic phosphates (Dunn et al., 1994). The vacuolar membrane of *S. cerevisiae* also contains Yvc1, a TRP-like calcium channel (Palmer et al., 2001; Denis and Cyert, 2002). Proper Ca^2+^ concentration in the endoplasmic reticulum (ER) organelle is critical to its functions; in *S. cerevisiae* it is maintained at 10 *μ*M (Strayle et al., 1999), well below the concentration found in this compartment in higher eukaryotes, where it is the main internal storage for calcium ions (Stefan C. J. et al., 2013). Cls2/Csg2, an ER-localized protein, was originally proposed to play a role in Ca^2+^ efflux from the ER (Beeler et al., 1994; Tanida et al., 1996). However, Csg2 is likely implicated in the mannosylation of the inositol-phosphoceramide (IPC), corroborating the evidences about the sphingolipid roles in regulating ionic channels (Birchwood et al., 2001; Montefusco et al., 2014). Although initially underestimated, the central role of the Golgi apparatus in calcium homeostasis and signaling is now well appreciated in eukaryotic cells (Pizzo et al., 2011), also in yeast cells (Miseta et al., 1999a; Wuytack et al., 2003). Pmr1, the budding yeast Golgi P-type Ca^2+^/Mn^2+^-ATPase, was the first member of the secretory pathway Ca^2+^-ATPase (SPCA) subfamily identified (Rudolph et al., 1989; Antebi and Fink, 1992; Sorin et al., 1997); its role is pivotal for the maintenance of proper Ca^2+^ levels in both the Golgi apparatus (Halachmi and Eilam, 1996; Miseta et al., 1999a), and the ER (Durr et al., 1998; Strayle et al., 1999). Early reports suggested that yeast mitochondria have little, if any, role in accumulating Ca^2+^ (Carafoli et al., 1970; Balcavage et al., 1973), and their Ca^2+^ levels appear to be comparable to those of cytosol (Jung et al., 2004; Niedzwiecka et al., 2018).

#### 2.1.3 The Calmodulin-Calcineurin Pathway

Calmodulin (CaM) is a highly conserved and ubiquitous Ca^2+^-binding protein that modulates the activity of many target enzymes, mainly in response to increasing intracellular Ca^2+^ concentrations (Cyert, 2001), which trigger distinct structural rearrangements, and modes of target activation (Nakashima et al., 2012; Ogura et al., 2012b; Ogura et al., 2012a; Ishida et al., 2002). The number of identified target proteins for mammalian CaM is, according to the Calmodulin Target Database, nearly 300 (Yap et al., 2000), whereas far fewer are known for the yeast CaM (Cyert, 2001). During stress responses, the yeast CaM functions primarily through the activation of a small fraction of its targets: the calmodulin-dependent protein kinases (encoded by *CMK1*, *CMK2*), and calcineurin. Calcineurin is a Ca^2+^/calmodulin-dependent serine/threonine-specific protein phosphatase, and represents the major Ca^2+^ signaling effector (Cyert and Thorner, 1992; Groppi et al., 2011; Li et al., 2011). Calcineurin regulates Ca^2+^ homeostasis and signaling both at the transcriptional level, for example by regulating the transcription of the Pmc1 encoding gene, and via the transcription factor Crz1 (Yoshimoto et al., 2002; Cyert, 2003), and at the post-translational level, for example by direct dephosphorylation of Vcx1. Some calcineurin targets involved in Ca^2+^ homeostasis are the HACS and the vacuolar Ca^2+^/H^+^ exchanger Vcx1 (Cunningham and Fink, 1996; Miseta et al., 1999b; Kingsbury and Cunningham, 2000).

### 2.2 Hypotonic Shock Response

Upon HTS, after the transient osmotic swelling, mammalian cells re-adjust their volume by a mechanism known as regulatory volume decrease (RVD) (Okada et al., 2001). In some cell types, RVD is accomplished by means of stretch-activated Ca^2+^ channels which mediate a rapid increase of the cytosolic Ca^2+^ concentration due to both Ca^2+^ influx and Ca^2+^ release from intracellular stores, which in turn is often the signal that triggers release of osmolytes, reducing osmotic gradients and helping volume regulation (Jakab et al., 2002). In addition, the HTS-induced activation of ion conducting pathways leads to profound changes in the plasma membrane potential, which determines the direction of the ion fluxes depending on the respective equilibrium potentials and the modulation of voltage-gated ionic channels.

Upon HTS, a large fraction of glycerol, an intracellular osmolyte, is released in yeast cells to the extracellular environment within 2–3 min through the activation of Fps1 channels (Tamas et al., 1999), which is inhibited neither by gadolinium, which instead completely blocks Ca^2+^ increase (Batiza et al., 1996), nor by membrane potential alterations (Kayingo et al., 2001). Fps1 was proposed as a mechanosensitive channel directly activated by the induced membrane stretch, whereas the known post-translational modifications probably fine-tune its activity under basal conditions (Ahmadpour et al., 2014).

Phosphoinositides are negatively charged membrane lipids that serve as versatile molecules involved in protein regulation, assembly of actin cytoskeleton, vesicle trafficking and Ca^2+^ signaling. Several phosphoinositides are substrates for phospholipases, thereby generating a number of products that serve as second messenger with biological functions on their own (Strahl and Thorner, 2007). For example, the plasma membrane lipid phosphatidylinositol 4,5-bisphosphate (PtdIns (4,5) P_2_) can be depleted by activation of phospholipase C (PLC), producing diacylglycerol (DAG) and the diffusible molecule Ins (1,4,5) P_3_ (IP_3_), which in mammals is a fundamental signaling molecule. In budding yeast, as in all eukaryotic cells, different phosphoinositide species are generated in a compartment-specific manner and, hence, can be regarded as distinct markers for each organelle (Odorizzi et al., 2000; Strahl and Thorner, 2007; Balla, 2013) and contribute to specific regulation of protein activity, such as ion channels (Hille et al., 2015). Upon HTS, *S. cerevisiae* cells hydrolyze plasma membrane PtdIns (4,5) P_2_ with a mechanism dependent on Plc1 but independent on the extracellular Ca^2+^ concentration. This process liberates IP_3_, which is then rapidly phosphorylated in IP_6_. In addition, another phosphoinositide, PtdIns4P, and is rapidly synthesized and then progressively consumed in the next minutes (Perera et al., 2004). Although a link between these dynamics and Ca^2+^ signaling has not been explored yet, it is tempting to suggest one. In fact, the rapid depletion of the plasma membrane signature lipid PtdIns-(4,5)-P_2_ could influence the activity of some channels. Flc2 is the best candidate, since it resides on plasma membrane and contains a putative lipid-binding domain. Channels localized on Golgi or ER could also be influenced by changes in phosphoinositides abundances: PtdIns4P transient increase could regulate Golgi channels, since PtdIns4P is the signature lipid of this organelle. Some Ca^2+^ regulation seems to be at stake in HTS because *PLC1* gene deletion, which abolishes PtdIns-(4,5)-P_2_ depletion and IP_6_ production, causes a greater increase in calcium influx after HTS compared to a wild type strain (Tisi et al., 2002). It is worth noting, however, that *PLC1* deletion does not affect PtdIns4P dynamics upon HTS (Perera et al., 2004).

The immediate and transient (∼2 min) cytosolic Ca^2+^ pulse (Batiza et al., 1996; Rigamonti et al., 2015) triggered by HTS in *S. cerevisiae* cells is generated both by influx from the extracellular medium and efflux from intracellular stores. An early study on yeast cells grown in synthetic medium reported that this increase was mediated by an instantaneous release of Ca^2+^ from intracellular stores and then sustained by influx of extracellular calcium. In fact, addition of an extracellular Ca^2+^ chelator, BAPTA, affected later stages of the response without affecting the initial, and rapid cytosolic Ca^2+^ rise (Batiza et al., 1996). Moreover, the same study showed that the HTS-induced Ca^2+^ response was dependent on both intensity of the shock and type of growth medium, the latter affecting also the pre-stimulus baseline [Ca^2+^]_cyt_. The increase of Ca^2+^ was inhibited in a dose-dependent manner by pre-treatment with gadolinium, a blocker of stretch-activated channels, and suggesting that the membrane stretching that occurs following HTS-induced cell swelling is directly sensed by Ca^2+^ channels (Batiza et al., 1996). In yeast cells grown in YPD—a complex, nutrient-rich medium—and challenged with HTS by diluting the medium with distilled water, an estimate of the initial rate of calcium increase at micromolar [Ca^2+^]_ext_ could be fitted by a Hill function, suggesting that the calcium increase in response to HTS was caused by the activation of a single channel or transporter located on the plasma membrane (Rigamonti et al., 2015).

The HTS response was also measured for mutants lacking proteins known to be involved in calcium signaling and homeostasis. *cch1*Δ mutants, lacking a functional HACS channel on the plasma membrane, responded to HTS with a higher calcium peak at all [Ca^2+^]_ext_ considered, suggesting a negative regulatory role for this protein during HTS. Mutants in other known influx pathways, on the other hand, and had a response similar to the wild-type. Calcium levels are affected in calcineurin mutants, suggesting that this Ca^2+^-dependent effector shapes the calcium signal during HTS through dephosphorylation of target transporters and/or by modulating their long-term expression. In addition to display altered resting calcium levels, mutants lacking calcineurin respond to HTS with a dramatically reduced peak. Flc2 was found to be involved in the HTS-induced calcium response, since deletion of *FLC2* increases the initial rate of the Ca^2+^ increase compared with wild-type, suggesting an inhibitory role of this protein on the channel that is activated by the HTS. Based on the experimental evidences described above, a model is proposed that includes only the essential players in the HTS-induced calcium response. In non signaling conditions, Ca^2+^ enters the cell through HACS channel and other unidentified influx pathways. A still unidentified mechanosensitive calcium channel is located on the plasma membrane and activated by the increased membrane tension caused by HTS. In addition, this channel appears to be negatively regulated by Flc2. Since the elevation of [Ca^2+^]_cyt_ is transient, some intracellular transporters must restore the steady-state levels of cytosolic Ca^2+^. This signal attenuation is probably performed by the Golgi-localized Pmr1, together with vacuolar-localized Pmc1 and Vcx1.

## 3 Methods

### 3.1 Model Definition and Simulation

The mathematical model of HTS response in *S. cerevisiae* was defined on the basis of available experimental evidences (Rigamonti et al., 2015), and can be conceptually divided in two modules:

1 the *biophysical module* describes the changes in volume and other cell parameters, such as turgor pressure and membrane tension. This module allows for quantitatively following all changes in the physical state of the cell depending on cytosolic and extracellular osmolarities. Such parameters, in turn, regulate the activity of some components of the biochemical module. In particular, stretch-activated channels open following a sudden increase of membrane tension, promoting Ca^2+^ diffusion through them;
2 the *biochemical module* describes all the relevant reactions that take place in the cell—or between the cell and the extracellular environment—during the HTS response. This module comprises two compartments: the cytosol and the extracellular environment.

The model was formalized as a system of coupled ODEs. Mass-action kinetics was used to model the physical interactions between Ca^2+^, calmodulin, and calcineurin. Most transport reactions were modeled by means of the Michaelis-Menten kinetics, as substantiated by previous studies (Ohsumi and Anraku, 1983; Wei et al., 1999; Takita et al., 2001; Teng et al., 2008).

Stretch-activated channels were modeled differently: they can be viewed as pores, whose opening probability depends on membrane tension. In particular, the opening probability of mechanosensitive channels was shown to follow a Boltzmann distribution (Gustin et al., 1988; Sukharev et al., 1999; Jiang and Sun, 2013). For the sake of simplicity, in this work the Boltzmann equation employs turgor pressure (note that membrane tensions can be calculated from turgor pressure using Laplace’s law for a thin-walled sphere (Gustin et al., 1988; Sackin, 1995)). The opening probability (*P*_open_) of mechanosensitive channels was thus formally defined as:

$$P_{\text{open}}=1-\frac{1}{1+e^{\frac{P-P_{\text{MS}}}{g_{\text{MS}}}}}$$ (1)

where *P* is turgor pressure, *P*_MS_ is the value of turgor pressure at which *P*_open_ is equal to 0.5, and *g*_MS_ is a slope parameter.

Since ions pass through the channel pore down their electrochemical gradient, we assume that calcium ions flow according to their concentration gradient. The calcium flux *j* is then given by:

$$j=P_{\text{open}}\cdot k\cdot \Delta c$$ (2)

where *k* is a rate parameter and Δ*c* is the Ca^2+^ concentration gradient across the membrane.

The model was simulated with COPASI (version: 4.19) (Hoops et al., 2006), using the LSODA algorithm (Petzold, 1983) with default settings. Simulation outputs consist in time traces of species concentrations over a period of 160 s, in line with the stress response duration of yeast cells. The model is available as an SBML Level 2 Version 5 file (ID MODEL2112030001) in the BioModels repository (Malik-Sheriff et al., 2020): https://www.ebi.ac.uk/biomodels/MODEL2112030001.

### 3.2 Parameter Estimation

Unknown parameters were estimated with COPASI (Hoops et al., 2006), using the available implementation of the Particle Swarm Optimization (PSO) algorithm (Kennedy and Eberhart, 1995). PSO is a global optimization algorithm based on the concept of “swarm intelligence”, which was shown to be effective in solving optimization problems characterized by multi-modal and noisy fitness landscapes, such as the ones related to the parameter estimation problem of biochemical systems (Nobile et al., 2018; Tangherloni et al., 2019; Besozzi et al., 2020).

To estimate the unknown parameters, simulations were fitted against available experimental time traces of [Ca_cyt_], measured in different experimental conditions in Rigamonti et al. (2015), including deletion mutants in key proteins of the HTS response and a wide range of extracellular Ca^2+^ concentrations. It is worth mentioning that, in the parameter estimation process, we took into account the well known fact that the intracellular calcium levels are kept in a narrow range despite wide variations in external conditions, thanks to calcium buffering and sensing and feedback mechanisms (Cunningham K. W. and Fink G. R., 1994). Thus, while reaction constants must be the same across different experiments, the concentrations of proteins bound to Ca^2+^ can vary. According to this line of reasoning, these parameters were not forced to be the same for all experiments. Since the model describes a stimulus response, the steady-state pre-stress conditions were also included in the parameter estimation process. Search ranges for all unknown parameters were set to be within biologically plausible numeric intervals.

## 4 Model Definition

### 4.1 Definition of the Biophysical Module

The biophysical module describes the variation of the physical parameters of the cell, such as the cell volume and turgor pressure. The following mathematical description of volume regulation under osmotic stress is a simplified version of a previously published model, which was carefully parameterized using hyperosmotic shock data (Schaber and Klipp, 2008).

#### 4.1.1 Volume

Assuming that volume changes are only due to water flow and not to solute flow, the total cell volume *V* (in L) can be defined as the sum of an osmotically active volume (water volume) *V*_os_ and an osmotically inactive volume (solid volume) *V*_b_:

$$V=V_{\text{os}}+V_{\text{b}},$$ (3)

with *V*_b_ assumed to be constant. The extracellular volume, *V*_ex_, is much greater than the volume occupied by the cell. Therefore, for the sake of simplicity, we assume that *V*_ex_ = *V* (0) ⋅ 1,000, where *V* (0) is the initial cell volume.

The water flow is driven by gradients of water potential and hydrodynamic potential (Griffin, 1981; Kleinhans, 1998), which can be formalized as:

$$\frac{dV}{dt}=\frac{d}{dt}\left(V_{\text{os}}+V_{\text{b}}\right)=\frac{dV_{\text{os}}}{dt}=-L_{p}A\left(P+\Delta \Pi_{n}-\sigma \Delta \Pi_{s}\right),$$ (4)

where *L*_*p*_ is the hydraulic conductivity (in dm⋅MPa^−1^⋅s^−1^), *A* is the cell surface area (in dm^2^) and *P* is the intracellular hydrostatic pressure exerted on the cell wall—i.e., the turgor (in MPa)—which equilibrates ΔΠ under steady-state conditions $\left(\frac{dV}{dt}=0\right)$ . ΔΠ is the osmotic pressure difference (in MPa) between the outside and the inside of the cell (subscripts *n* and *s* denote non-permeable and permeable solutes, respectively), while the dimensionless parameter *σ* is the reflection coefficient, which depends on the solute permeability (Kedem and Katchalsky, 1958). In *S. cerevisiae*, the main permeable solute is glycerol (Reed et al., 1987), which is released by Fps1 channels (Luyten et al., 1995). When water and solutes are transported by different channels, the reflection coefficient has been shown to be:

$$\sigma =1-\frac{k_{s}\bar{V}}{RTL_{p}},$$ (5)

where $\bar{V}$ is the partial molar volume (in m^3^⋅mol^−1^) of the solute, *k*_*s*_ is the membrane solute permeability (in dm⋅s^−1^), *R* is the gas constant (in *J*⋅mol^−1^⋅K^−1^) and *T* is the temperature (in K) (Kedem and Katchalsky, 1958; Kleinhans, 1998). Since the only solute considered here is glycerol, *σ* is approximately equal to 1 at room temperature^1^, and thus:

$$\frac{dV_{\text{os}}}{dt}=-L_{p}A\left(P+\Delta \Pi_{n}-\Delta \Pi_{s}\right),$$ (6)

with $A≔A\left(t\right)={\left(36\pi \right)}^{\frac{1}{3}}V{\left(t\right)}^{\frac{2}{3}}$ , since the cell has a roughly spherical shape.

The van’t Hoff law can be used to express the osmotic pressure in terms of concentration of osmotically active molecules: ΔΠ = *c*_*PC*_ *RT*Δ*c*, where Δ*c* is the concentration and *c*_*PC*_ is a conversion factor relating concentrations in M to pressures in MPa. Thus, Eq. 6 can be written as:

$$\frac{dV_{\text{os}}}{dt}=-L_{p}A\left(P+c_{PC}RT\left(\left[Osm_{e}\right]-\left[Osm_{i}\right]\right)\right),$$ (7)

where Δ*c* is substituted with [*Osm*_*e*_] − [*Osm*_*i*_], where [*Osm*_*e*_] and [*Osm*_*i*_] are extracellular and intracellular concentration (in *μ*mol⋅L^−1^) of osmotically active molecules, respectively. If Eq. 7 is initially at steady-state $\left(\frac{dV_{\text{os}}}{dt}=0\right)$ , the internal osmolarity can be estimated as a function of the initial turgor pressure *P*_0_ and the initial external osmolarity *Osm*_*e*_ (0) as:

$$\left[Osm_{i}\right]\left(0\right)=\left[Osm_{e}\right]\left(0\right)+\frac{P_{0}}{c_{PC}RT}.$$ (8)

Both intracellular and extracellular osmolarities are the sum of concentrations of permeable solutes (glycerol) and non-permeable solutes. The extracellular osmolarity is thus:

$$\left[Osm_{e}\right]=c_{n}^{e}+\left[Gly_{e}\right]-\left[Gly_{e}\right]\left(0\right),$$ (9)

where $c_{n}^{e}$ is the extracellular concentration of non-permeable solutes, which varies in time according to the applied stimulus (explained below), while [*Gly*_*e*_] is the extracellular glycerol concentration. The initial extracellular glycerol concentration ([*Gly*_*e*_](0)) is assumed to be 1,000 times lower than the initial intracellular glycerol concentration (that is, [*Gly*_*e*_](0) = [*Gly*_*i*_](0)/1,000), providing a gradient for glycerol efflux from the cell. All concentrations are expressed in *μ*mol⋅L^−1^.

The intracellular osmolarity, accounting for cell volume variation, is defined as:

$$\left[Osm_{i}\right]=\left[Gly_{i}\right]+\frac{c_{n}^{i}V_{os}\left(0\right)}{V_{os}},$$ (10)

where [*Gly*_*i*_] is the intracellular glycerol concentration, $c_{n}^{i}=\left[Osm_{i}\right]\left(0\right)-\left[Gly_{i}\right]\left(0\right)$ is the concentration of non-permeable solutes, while *V*_*os*_ and *V*_*os*_(0) are, respectively, the cytosolic volume and the cytosolic volume at *t* = 0. Concentrations are expressed in *μ*mol⋅L^−1^, while volumes are expressed in *μ*m^3^.

#### 4.1.2 Turgor Pressure

The water potential gradient maintained by all cells across their membrane is balanced by a hydrostatic pressure called turgor. In walled cells, turgor pressure causes the cell membrane to exert a force on the cell wall, which expands due to its elasticity. The elastic-theory of turgor pressure states that the change in turgor pressure *P* is proportional to a relative change in cell volume:

$$dP=-\epsilon \frac{dV}{V},$$ (11)

where the proportionality factor *ϵ* is called volumetric elastic modulus, or Young’s modulus (in MPa).

The dependence of turgor pressure on volume can be deduced by integration:

$$\int_{V_{0}}^{V}dP\left(V\right)=-\int_{V_{0}}^{V}\epsilon \frac{1}{V}\;\;\Leftrightarrow \;\;P\left(V\right)-P\left(V_{0}\right)=-\epsilon \ln\left(\frac{V}{V_{0}}\right).$$ (12)

By defining *V*_0_ as the volume when turgor becomes zero, the turgor pressure can be expressed as a function of volume:

$$\left\{\begin{array}{cc} P\left(V,V_{0},\epsilon \right)=-\epsilon \ln\left(\frac{V}{V_{0}}\right)\; & \text{for}\;V\geq V_{0},\\ 0\; & \text{for}\;V<V_{0}. \end{array}\right.$$ (13)

It is known that glycerol efflux and synthesis is tightly regulated according to environmental conditions (Talemi et al., 2016). However, given the small time-scales considered here, internal glycerol concentration is assumed to be constant.

#### 4.1.3 Hypotonic Shock

Hypotonic shock is applied to the cell by diluting the medium with distilled water, thus decreasing its osmolarity. This dilution is modeled as follows:

$$\left\{\begin{array}{cc} c_{n}^{e}=c_{0}^{e}\; & \text{for}\;t<t_{off},\\ c_{n}^{e}=\left(c_{0}^{e}-\frac{c_{0}^{e}}{d}\right)e^{\frac{t_{off}-t}{t_{m}}}+\frac{c_{0}^{e}}{d}\; & \text{otherwise,} \end{array}\right.$$ (14)

where $c_{0}^{e}$ is the initial extracellular osmolarity (in *μ*mol⋅L^−1^), *d* is the diluting factor, *t* is the simulation time instant (in s), *t*_*off*_ (in s) is the instant when dilution is applied, and *t*_*m*_ is the mixing time (in s) that regulates the speed of dilution.

### 4.2 Definition of the Biochemical Module

The concentration of calcium ions in the cytosol changes due to fluxes across different channels and transporters. The cytosol is also provided with calmodulin—a protein involved in the binding and sensing of calcium ions (Cyert, 2001)—that can activate calcineurin, the main calmodulin effector. HTS is applied by diluting the medium in which cells grow with distilled water, which has also the effect of reducing the availability of calcium ions. Since the extracellular volume is way larger than the volume occupied by all cells, the reduction of extracellular calcium ions caused by the cell uptake can be neglected. Therefore, the extracellular Ca^2+^ concentration depends only on dilution factor and mixing time (see Eq. 14):

$$\left\{\begin{array}{cc} \left[{\text{Ca}}_{\text{ex}}\right]=\left[{\text{Ca}}_{\text{ex}}\right]\left(0\right)\; & \text{for}\;t<t_{off},\\ \left[{\text{Ca}}_{\text{ex}}\right]=\left(\left[{\text{Ca}}_{\text{ex}}\right]\left(0\right)-\frac{\left[{\text{Ca}}_{\text{ex}}\right]\left(0\right)}{d}\right)e^{\frac{t_{off}-t}{t_{m}}}+\frac{\left[{\text{Ca}}_{\text{ex}}\right]\left(0\right)}{d}\; & \text{otherwise,} \end{array}\right.$$ (15)

with all concentrations expressed in nM, and *t*, *t*_*off*_ and *t*_*m*_ expressed in seconds.

The rate of change of the Ca^2+^ concentration in the cytosol can be written as the sum of fluxes of the relevant channels and transporters (described below):

$$\frac{d\left[{\text{Ca}}_{\text{cyt}}\right]}{dt}=j_{\text{IN}}+j_{\text{Cch}1}+j_{\text{MS}}-j_{\text{Pmr}1}-j_{\text{Vcx}1}-j_{\text{Pmc}1},$$ (16)

where the *j*s are Ca^2+^ fluxes (in nM⋅s^−1^) across calcium channels and transporters.

Very often, channels and transporters show kinetics that can be described with the Michaelis-Menten equation (i.e. the transporter saturates at high substrate concentrations) (Christopher, 2002; Weijiu, 2012; Fridlyand et al., 2003). Indeed, this is the case for the intracellular transporters Pmr1, Vcx1, Pmc1 (see references in Table 2). Pmr1 indirectly replenishes the endoplasmic reticulum with Ca^2+^, while Vcx1 and Pmc1 are responsible for its sequestration into the vacuole. Ca^2+^ enters the cell through the plasma membrane-located HACS channel and other transporters whose molecular identities are yet unknown (Batiza et al., 1996; Locke et al., 2000; Tisi et al., 2002; Cui et al., 2009a). Here, the influx associated with this unknown transport is simply called *j*_IN_—to recall its function—and is assumed to have a Michaelis-Menten kinetics.

Experimental evidences strongly suggest that the increase of cytosolic Ca^2+^ in cells challenged with HTS is caused by the opening of a MS channel on the plasma membrane (Batiza et al., 1996; Rigamonti et al., 2015). This calcium influx pathway has not been molecularly identified yet, and it is here denoted by *j*_MS_. The equations describing the fluxes are:

$$j_{\text{IN}}=\frac{v_{\text{IN}}\;\left[{\text{Ca}}_{\text{ex}}\right]}{k_{\text{IN}}+\left[{\text{Ca}}_{\text{ex}}\right]},$$ (17)

$$j_{\text{Pmr}1}=\frac{v_{\text{Pmr}1}\;\left[{\text{Ca}}_{\text{cyt}}\right]}{k_{\text{Pmr}1}+\left[{\text{Ca}}_{\text{cyt}}\right]},$$ (18)

$$j_{\text{Vcx}1}^{0}=\frac{v_{\text{Vcx}1}\;\left[{\text{Ca}}_{\text{cyt}}\right]}{k_{\text{Vcx}1}+\left[{\text{Ca}}_{\text{cyt}}\right]},$$ (19)

$$j_{\text{Pmc}1}=\frac{v_{\text{Pmc}1}\;\left[{\text{Ca}}_{\text{cyt}}\right]}{k_{\text{Pmc}1}+\left[{\text{Ca}}_{\text{cyt}}\right]},$$ (20)

$$j_{\text{MS}}^{0}=P_{\text{open}}\;k_{\text{MS}}\;\left(\left[{\text{Ca}}_{\text{ex}}\right]-\left[{\text{Ca}}_{\text{cyt}}\right]\right),$$ (21)

$$j_{\text{Cch}1}^{0}=k_{\text{Cch}1}\;\left(\left[{\text{Ca}}_{\text{ex}}\right]-\left[{\text{Ca}}_{\text{cyt}}\right]\right),$$ (22)

where *P*_open_ is the opening probability of the MS channel (see below), *k*_Cch1_ and *k*_MS_ are rate parameters (in s^−1^), *v*s. are rate constants (in nM⋅s^−1^), and all other *k*s are Michaelis constants (in nM).

The opening probability of MS channels follows a Boltzmann distribution (Gustin et al., 1988; Sukharev et al., 1999; Jiang and Sun, 2013). MS channels are gated by membrane tension (Gustin et al., 1988; Sackin, 1995) but here, for the sake of simplicity, turgor pressure is used instead (see Section 3.1 and Eq. 1 for a justification). The opening probability of the MS channel is then:

$$P_{\text{open}}=1-\frac{1}{1+e^{\frac{P-P_{\text{MS}}}{g_{\text{MS}}}}},$$ (23)

where *P* is the cell turgor pressure in MPa (see Eq. 13), *P*_MS_ is the turgor pressure (in MPa) at which *P*_open_ is equal to 0.5, and *g*_MS_ is a slope parameter (in MPa).

#### 4.2.1 Feedback Regulation

Inside the cytosol, yeast calmodulin binds three Ca^2+^ ions with high cooperativity (Davis et al., 1986; Nakashima et al., 1999):

$$\text{CaM}+3{\text{Ca}}^{2+}\underset{k_{m}^{-}}{\overset{k_{m}^{+}}{⇋}}\text{CaMCa}.$$ (24)

By using the law of mass action, the rate equation for Ca^2+^-bound calmodulin can be formalized as:

$$\frac{d\left[\text{CaMb}\right]}{dt}=k_{m}^{+}\left(\left[\text{CaMt}\right]-\left[\text{CaMb}\right]\right){\left[{\text{Ca}}_{\text{cyt}}\right]}^{3}-k_{m}^{-}\left[\text{CaMb}\right],$$ (25)

where [CaMb] is the concentration of Ca^2+^-bound calmodulin (in nM), CaMt is the total calmodulin concentration (in nM), $k_{m}^{+}$ and $k_{m}^{-}$ are the forward and backward rate constants (in nM^−4^⋅s^−1^ and s^−1^, respectively).

Calcineurin, a protein phosphatase, is activated upon binding with the Ca^2+^-bound calmodulin. By the law of mass action we can state that:

$$\frac{d\left[\text{CaNb}\right]}{dt}=k_{n}^{+}\left(\left[\text{CaNt}\right]-\left[\text{CaNb}\right]\right)\left[\text{CaMb}\right]-k_{n}^{-}\left[\text{CaNb}\right],$$ (26)

where [CaNb] is the concentration of calmodulin-bound calcineurin (in nM), CaNt is the total calcineurin concentration (in nM), $k_{n}^{+}$ and $k_{n}^{-}$ are the forward and backward rate constants (in nM^−2^⋅s^−1^ and s^−1^, respectively).

Following HTS, calcium ions enter the yeast cell through MS channels located on the plasma membrane. After the initial rise in Ca^2+^, the signal dissipation observed in the successive seconds must be the result of either an increased activity of one of the intracellular transporters or a feedback inhibition on the MS channels. Since no positive regulation is known for any of the relevant transporters, in the present model the latter mechanism is assumed. This assumption is supported by circumstantial evidence suggesting that the yeast MS channel interacts with a homologous of TRP proteins (Rigamonti et al., 2015). TRP proteins form tetrameric ion channels which frequently interact with—and are inhibited by—calmodulin (Rhoads and Friedberg, 1997; Zhu, 2005). It is also known that activated calcineurin post-transcriptionally inhibits Vcx1 and HACS activity (Cunningham and Fink, 1996; Miseta et al., 1999b; Locke et al., 2000). The following equations are thus used to model the feedback inhibition:

$$j_{\text{Cch}1}=\frac{j_{\text{Cch}1}^{0}}{1+k_{I\text{Cch}1}\;\left[\text{CaNb}\right]},$$ (27)

$$j_{\text{MS}}=\frac{j_{\text{MS}}^{0}}{1+k_{I\text{MS}}\;\left[\text{CaMb}\right]},$$ (28)

$$j_{\text{Vcx}1}=\frac{j_{\text{Vcx}1}^{0}}{1+k_{I\text{Vcx}1}\;\left[\text{CaNb}\right]},$$ (29)

where *k*_*I*Cch1_, *k*_*I*MS_ and *k*_*I*Vcx1_ are inhibition constants (in nM^−1^).

### 4.3 Estimation of the Unknown Parameters

**FIGURE 2:**
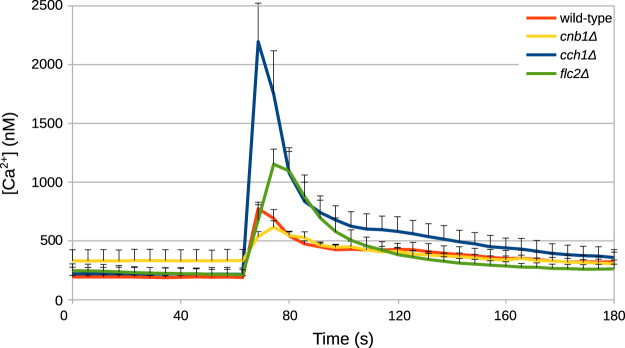
Example of time traces of cytosolic Ca^2+^ concentration in *S. cerevisiae* cells challenged with HTS. HTS was applied by diluting the growth medium with four volumes of distilled water at *t* =60. The final concentration of Ca^2+^ in the medium was 5.9 *μ*M. Time traces were taken from Rigamonti et al. (2015) and from Tisi R., unpublished results.

Unknown parameters of the model were fitted against time traces of cytosolic Ca^2+^ measurements from HTS experiments conducted by Rigamonti et al. (2015) and by R. Tisi, unpublished results (Figure 2), as described in Materials and Methods. In these experiments, wild-type *S. cerevisiae* cells were grown in YPD medium, which has an estimated osmolarity of 0.26 Osm/L (Schaber et al., 2010). Specifically, the parameters of the biophysical module (Table 1) were set to reflect those experimental conditions.

**TABLE 1:** List of parameters of the biophysical module.

Parameters	Value	Unit	References
*V*_*b*_	20.5 ⋅ 10^–15^	L	Schaber et al. (2010)
*V* (0)	50 ⋅ 10^–15^	L	
*V*_*ex*_	1,000 ⋅ *V* (0)	L	
*V*_*os*_(0)	*V* (0) − *V*_*b*_	L	
*L*_*p*_	1.3 ⋅ 10^–7^	dm⋅MPa^−1^⋅s^−1^	Talemi et al. (2016)
*P*_0_	0.61	MPa	Schaber et al. (2010)
*c*_*PC*_	10^–9^	–	
*R*	8.314	*J*⋅mol^−1^⋅K^−1^	
*T*	303.15	K	
$c_{0}^{e}$	260,000	*μ*mol ⋅ L^−1^	Schaber et al. (2010)
*D*	variable	–	Rigamonti et al. (2015)
*t*_*off*_	30	s	Rigamonti et al. (2015)
*t*_*m*_	10	s	
[*Gly*_*i*_]	180,000	*μ*mol ⋅ L^−1^	Schaber et al. (2010)
*ϵ*	14.3	MPa	

In addition to the wild-type strain, we simulated three mutant strains: *cnb1*Δ, lacking functional calcineurin; *flc2*Δ, lacking a putative TRP-like channel subunit; and *cch1*Δ, lacking a functional HACS channel. Within the model, a mutant can be simulated by setting to zero the parameter associated with the function carried out by the removed gene. Hence, the *cnb1*Δ strain was obtained by setting to zero the total amount of calcineurin in the cell (CaNt), and the *cch1*Δ by setting to zero the rate parameter *k*_Cch1_. However, these parameter settings were not sufficient to reproduce the behavior displayed by the three mutants. Thus, we performed additional parameter estimations exploiting available experimental time traces of the mutant strains (from Rigamonti et al. (2015) and R. Tisi, unpublished results, Figure 2).

In particular, *PMC1* expression strongly depends on calcineurin (Cunningham and Fink, 1996), but the model can not automatically adjust *PMC1* expression in the *cnb1*Δ mutant, since it lacks any description of transcriptional processes. To take into account the reduction of *PMC1* expression in this mutant, we performed a separate parameter estimation to infer the value of the *v*_Pmc1_ parameter (i.e., the *V*_max_ parameter of a Michaelis-Menten equation (Eq. 20), which is proportional to the enzyme abundance) in the *cnb1*Δ model. By doing so, it was possible to assess the reduction of Pmc1 transporters in this mutant with respect to the wild-type. We argue that the estimated value of *v*_Pmc1_ for the *cnb1*Δ mutant could be regarded as an estimate of the basal expression level of the Pmc1 protein, when Cnb1 is not stimulating the *PMC1* gene transcription. Thus, we used the same value of *v*_Pmc1_ to simulate the *cch1*Δ model. In fact, in order to maintain a physiological Ca^2+^ level when the Ca^2+^ income is lower, a novel steady state balance has to be achieved, where Pmc1 abundance has to be adjusted so that Ca^2+^ remains available to be provided to the secretory pathway as well. This is achieved by finely tuning the homeostasis regulatory circuit, through calcineurin transcriptional control on *PMC1* gene, leading to a Pmc1 activity nearby the basal level estimated for the *cnb1*Δ mutant.

Adopting a similar approach to the one described above, we estimated the value of *k*_MS_ for the *flc2*Δ strain, in order to account for the observed increased activity of the MS channel in this strain (Rigamonti et al., 2015). It is not known whether the increased activity of this channel is due to an increased channel abundance or simply to an increased channel activity. In any case, the rate parameter *k*_MS_ is—like the *V*_max_ of a Michaelis-Menten equation—proportional to the number of channels.

Analysis of the parameter estimation results led to the conclusion that the model could be simplified without affecting the simulations. In particular, removal of (*a*) the feedback inhibition on the HACS channel, mediated by calcineurin (Eq. 27), and of (*b*) the influx pathway that was called “IN” (Eq. 17), did not cause any significant difference on the simulation outcomes. Therefore, the feedback on HACS and the *j*_IN_ influx were removed and all simulations and parameter values reported here are related to this simplified model. Table 2 lists all the parameters used for the wild-type model, while Table 3 contains only the values that changed depending on the strain.

**TABLE 2:** List of parameters of the wild-type calcium model.

Parameters	Value	Range	Unit	References
[Ca_cyt_](0)	100		nM	Cunningham and Fink, (1994a)
[Ca_ex_](0)	29,500		nM	Rigamonti et al. (2015)
*t*_*m*_	9.1	1—10	s	
*v*_Vcx1_	2,820,420	100—5 ⋅ 10^6^	nM ⋅ s^−1^	
*v*_Pmc1_	280,870	100—5 ⋅ 10^5^	nM ⋅ s^−1^	
*v*_Pmr1_	813	100—5 ⋅ 10^5^	nM ⋅ s^−1^	
*k*_MS_	132,184	0.1—10^6^	s^−1^	
*k*_Cch1_	0.37	0.1—10^6^	s^−1^	
*k*_Pmr1_	70		nM	(Sorin et al., 1997; Wei et al., 1999)
*k*_Vcx1_	100,000		nM	Ohsumi and Anraku, (1983)
*k*_Pmc1_	4,300		nM	Takita et al. (2001)
*P*_MS_	0.76	0.6—0.8	MPa	
*g*_MS_	0.039	0.00001—1	MPa	
$k_{\text{m}}^{+}$	1.8 ⋅ 10^−14^	10^−20^—0.01	nM^−4^⋅s^−1^	
$k_{\text{m}}^{-}$	$k_{\text{m}}^{+}\cdot$ 9,000		s^−1^	Starovasnik et al. (1993)
$k_{\text{n}}^{+}$	0.1	10^−8^—10	nM^−2^⋅s^−1^	
$k_{\text{n}}^{-}$	1,000	10^−4^—10^4^	s^−1^	
[CaMt]	2,600		nM	Ho et al. (2018)
[CaNt]	310		nM	Ho et al. (2018)
*k*_*I*MS_	26,395	100—10^8^	nM^−1^	
*k*_*I*Vcx1_	9,348,540	100—10^8^	nM^−1^	

Known parameters were fixed, while parameters that needed to be estimated were allowed to change inside the values shown in the “Range” column. Simulations of the mutant strains were done with the parameters listed here, except for those listed in Table 3.

**TABLE 3:** List of parameters that change depending on the modeled mutant strain. All other parameters were kept as in the wild-type model (Table 2).

	*v*_Pmc1_ (nM⋅s^−1^)	*k*_Cch1_ (s^−1^)	*k*_MS_ (s^−1^)	[CaNt] (nM)
wild-type	280,870	0.37	132,184	310
*flc2*Δ	280,870	0.37	238,519	310
*cch1*Δ	41,079	0	132,184	310
*cnb1*Δ	41,079	0.37	132,184	0

## 5 Results

### 5.1 Model Simulations

**FIGURE 3:**
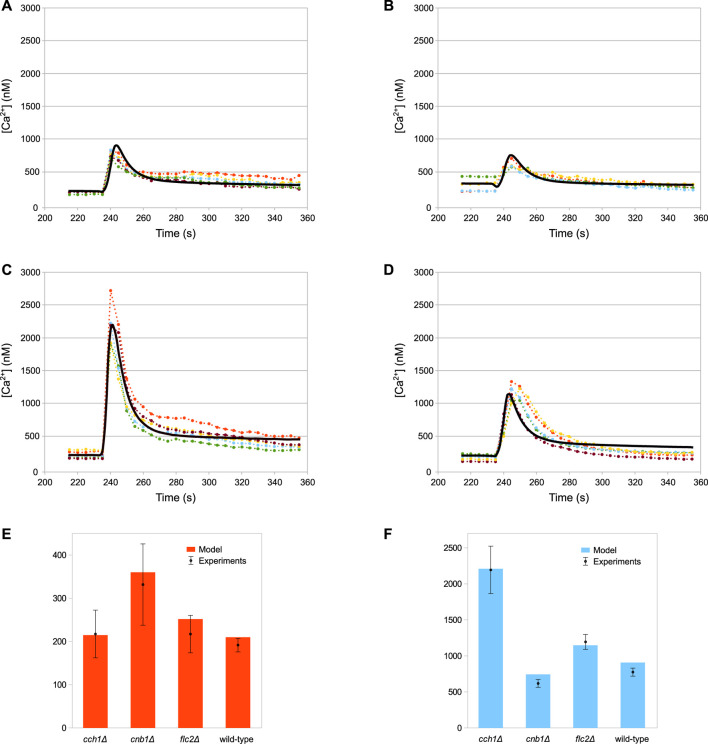
Comparison of simulation outcomes and experimental measurements. In Figures **(A–D)** the dotted lines represent experimental measurements and different colours indicate replicates, while the black lines represent model simulations. **(A)** Wild-type. **(B)** *cnb1*Δ. **(C)** *cch1*Δ. **(D)** *flc2*Δ. **(E)** Baseline cytosolic Ca^2+^ concentration (nM). **(F)** Max peak cytosolic Ca^2+^ concentration (nM). HTS was applied to the cells at 235 s by adding four volumes of distilled water to the medium **(E,F)** Plots constructed using the same data shown in **(A–D)**; bars represent standard deviations.

The simulations of the full model of HTS response correctly reproduce the dynamics of the Ca^2+^ transients (Figures 3A–D), as well as the steady-state levels and peak values of cytosolic Ca^2+^ (Figures 3E,F) of both wild-type and all mutant strains.

**FIGURE 4:**
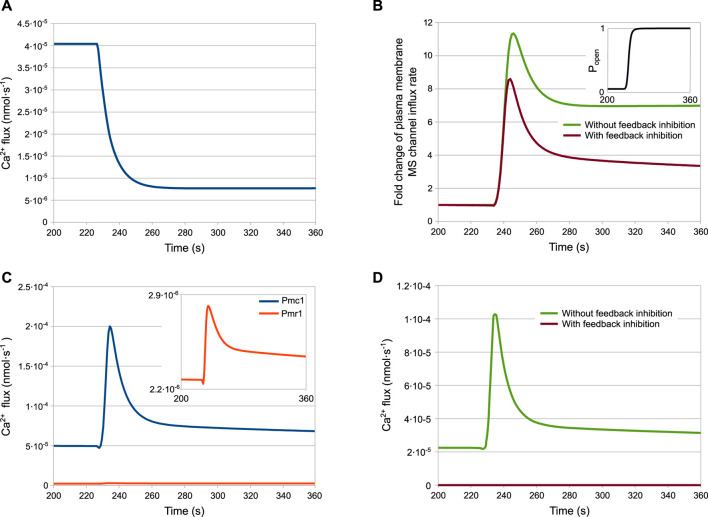
Fluxes involved in the HTS response of the wild-type strain. **(A)** HACS flux. **(B)** Effect of calmodulin inhibition on MS channels. **(C)** Pmc1 and Pmr1 fluxes. **(D)** Effect of calcineurin inhibition on Vcx1 **(A–D)** Ca^2+^ fluxes through channels and transporters as predicted by the model (parameters are given in Table 2) **(B,D)** Fluxes with or without inhibition are depicted by plotting *j*_MS_ and $j_{\text{MS}}^{0}$ for MS channel, and *j*_Vcx1_ and $j_{\text{Vcx}1}^{0}$ for the Vcx1 transporter. Inset in **(B)** shows how the opening probability of MS channels changes with time (note that it is not equal to zero before the stimulus). Fluxes in **(B)** are normalised to their steady-state values to better appreciate the long-term effect of channel inhibition.

The results of the simulations for the wild-type strain are shown in Figure 4. According to the model, after the HTS the HACS activity decreases due to the sudden shortage of the extracellular Ca^2+^ (Figure 4A), while the MS channels on the plasma membrane open rapidly (Figure 4B, inset). The cytosolic Ca^2+^-dependent inhibition on the MS channels provides a way to decrease the activity of this channel, thus helping to restore low intracellular calcium levels after the stimulus. In fact, the dynamics reported in Figure 4B shows that only in the presence of feedback inhibition the flux through this channel is still decreasing at time *t* > 280s. These outcomes also suggest that the main pump responsible for signal attenuation is Pmc1, the Ca^2+^-ATPase located on the vacuole, while Pmr1 and Vcx1 have a negligible role in this respect (Figures 4C,D). In particular, while the predicted rate of Ca^2+^ sequestration by Pmr1 is predicted to be consistently slow, the Vcx1 activity is kept down by the feedback inhibition mediated by calcineurin (Figure 4D).

**FIGURE 5:**
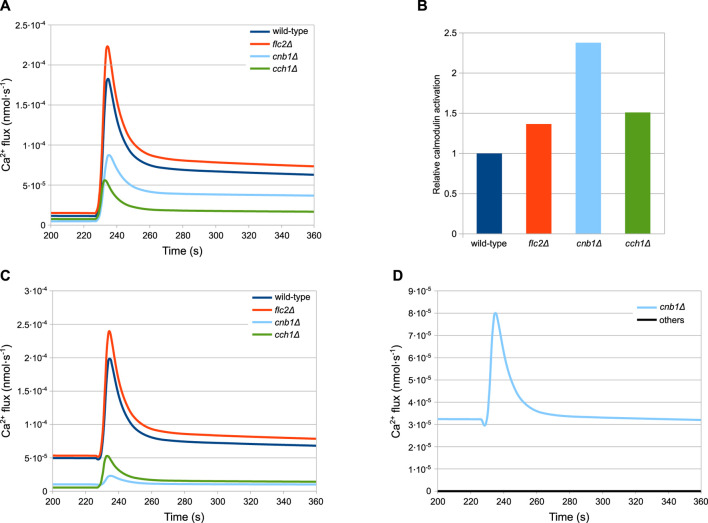
Simulations showing the main differences among the considered strains. **(A)** MS influx. **(B)** Calmodulin activation. **(C)** Pmc1 flux. **(D)** Vcx1 flux **(A,C,D)** Fluxes through the most relevant channels. **(B)** Calmodulin activation, shown relative to the wild-type level, before HTS is applied. Time traces of calcineurin are similar and are not shown for the sake of figure readability. **(D)** Vcx1 fluxes of all mutants but *cnb1*Δ are overlapping and therefore are coloured black.

As described above, the peak response of *cch1*Δ mutants—lacking functional HACS—is considerably higher than in the wild-type strain (Figure 3F). According to the model, the peak difference is not ascribable to an increased activity of the MS channels in this strain with respect to the wild-type. In fact, in this mutant, Ca^2+^ fluxes through both MS channels and Pmc1 are considerably lower than both wild-type and *flc2*Δ strains (Figures 5A,C). This behaviour is the result of the reduced *PMC1* expression (Table 3):

Since Pmc1 is the main transporter responsible for Ca^2+^ sequestration from the cytosol, reducing its activity increases the level of Ca^2+^, which in turn inhibits the MS channels through the activated calmodulin (Figure 5B). This feedback process ensures that the steady-state calcium levels are comparable to those of the wild-type. However, the reduced activity of Pmc1 causes a higher peak when the MS channels open following HTS. Rigamonti *et al.* suggested that the increased Ca^2+^ peak in *flc2*Δ is caused by the removal of an inhibitory effect on the MS channels (Rigamonti et al., 2015). To test this hypothesis, during the parameter estimation process we let the *k*_MS_ parameter of *flc2*Δ mutant assume different values compared to other strains (Table 3). The simulations show that, in line with this, a difference in the MS channels rate compared to the wild-type is sufficient to explain the increased peak observed during HTS (Figure 5A).

The *cnb1*Δ mutants show an increased steady-state level of cytosolic Ca^2+^ and a lower peak compared to all other strains (Figure 3). As described in Section 4.3, for this mutant cells we estimated the reduction of *PMC1* expression. Our model predicts that Pmc1 abundance is about 16 times lower compared to the wild-type, which is counterbalanced by the increased activity of Vcx1 that is no longer inhibited by calcineurin. This compensation mechanism, however, fails to fully replace the decreased activity of Pmc1, and thus the mutant displays higher steady-state calcium levels. The higher calcium levels result in more activated calmodulin (Figure 5B), which in turn inhibits the MS channels leading to a lower peak (Figure 5A).

**FIGURE 6:**
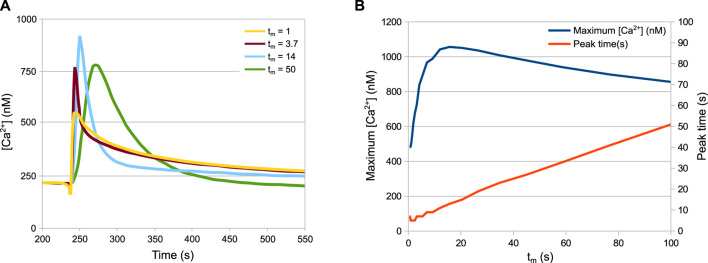
Behaviour of the system depending on the speed of the HTS. **(A)** Ca^2+^ peaks in response to different dilution speeds. **(B)** Maximum Ca^2+^ values and time of maximum Ca^2+^ values depending on the dilution speed. The *t*_*m*_ parameter is inversely proportional to the speed of dilution. Increasing the speed of dilution increases the speed of response but not necessarily the peak height.

Figure 6 shows a prediction of HTS response in not yet tested experimental conditions, since the technical difficulty in achieving a tight control of the speed of dilution could not be overcome. Variability in the speed and shape of the signal was observed in different experiments, particularly when an automatic injector was not applied, thus we decided to investigate the effect of this parameter variation with *in silico* experiments. The *t*_*m*_ parameter of the model defines the mixing time, i.e. the speed of dilution. The simulations of the wild-type strain with different mixing times produces an unexpected pattern (Figure 6A). In particular, while the speed of the response turns out to be linearly dependent on the mixing time (Figure 6B, red curve), the peak Ca^2+^ value is not. The peak values grow with the mixing time until a maximum at *t*_*m*_ = 15s, before decreasing again (Figure 6B, blue curve).

The model also predicts that the inactivation of the MS channels—simulated by setting to zero the parameter *k*_MS_—would reduce the steady-state cytosolic Ca^2+^ concentration from 215 to 160 nM.

## 6 Discussion

*S. cerevisiae* cells have to adapt to changes in growth conditions that arise both naturally, in the environment where they live, and artificially, during human exploitation. All cells sense extracellular osmolarity and fine-tune their biophysical parameters to ensure survival (Pedersen et al., 2011). Generation of a Ca^2+^ transient in response to HTS seems to be an ubiquitous phenomenon shared by yeast, plant and mammalian cells, and it is caused by the opening of MS channels (Sachs and Morris, 1998; Cox et al., 2013). In the yeast *S. cerevisiae*, many proteins involved in Ca^2+^ handling have been identified (Cui et al., 2009b; Tisi et al., 2016). However, only recently the specific roles of each channel or transporter in building, attenuating and shaping the observed Ca^2+^ dynamics are being investigated. The model presented here can help in bridging the gap between genetic analyses and phenotypic observations regarding Ca^2+^ signaling in yeast cells.

Simulations of the biophysical module alone showed that the volume of yeast cells increases only by a few percent relative to the pre-stress volume. This is in contrast to non-walled cells, whose volume can increase up to 50% (Weskamp et al., 2000). This difference is not surprising, as yeast cells must face harsher and unpredictable osmotic conditions than single cells inside a multicellular organism, and have therefore evolved a rigid cell wall.

In *S. cerevisiae* cells the vacuole is the main organelle for Ca^2+^ storage (Dunn et al., 1994), while in mammalian cells this role is played by the ER (Montero et al., 1995; Strayle et al., 1999). In fact, our simulations shows that most of the Ca^2+^ is transported into the vacuole. In all modeled strains, with the exception of *cnb1*Δ, Pmc1 appears to be the main vacuolar transporter. As expected, removal of calcineurin have opposite effects on vacuolar transporters: it reduces the expression of *PMC1*, but it increases the activity of Vcx1. However, our simulations suggest that the loss of Pmc1 activity is not entirely counterbalanced by Vcx1, and thus the cytosolic calcium level is higher than in the wild-type. These results are in accordance with calcium accumulation measurements showing that *pmc1*Δ mutants accumulate only 20% calcium compared to wild-type (Cunningham and Fink, 1996). In addition, the predictions made by our model—namely, that Vcx1 has a small role in calcium accumulation unless calcineurin is removed—are supported by the fact that *vcx1*Δ mutants accumulate the same amount of calcium as the wild-type, and that Vcx1 has a larger role in Ca^2+^ sequestration in *cnb1*Δ mutants (Cunningham and Fink, 1996). Pmr1 is important for the maintenance of proper calcium levels in the ER, where the steady-state free concentration is only 10 *μ*M (Strayle et al., 1999). Our simulations suggest also that Pmr1 has a marginal role during the HTS response, as well as in maintaining low steady-state calcium level into the cytosol. However, no definitive comparison can be drawn between model predictions and experimental results because *pmr1*Δ mutants display pleiotropic defects that are probably direct consequences of ER calcium depletion (Rudolph et al., 1989; Antebi and Fink, 1992).

Yeast cells are equipped with a variety of calcium influx pathways, each with seemingly different functions (Eilam and Othman, 1990; Iida et al., 1994; Fischer et al., 1997; Muller et al., 2003). Our model initially included three calcium entry pathways: HACS (the high affinity calcium system composed of at least Cch1 and Mid1), MS channels, and another calcium influx of unknown molecular identity. The presence of MS channels on the yeast plasma membrane was demonstrated by patch-clamp experiments (Gustin et al., 1988), but their molecular identities are still unknown. Parameter estimation revealed that the additional influx (here called “IN”) was unnecessary in this model, and that HACS, and the MS channels alone are sufficient to explain the observed data. The removal of “IN”, which was the only active transporter on the plasma membrane introduced in the model, implies that in the simulations Ca^2+^ enters the cell only by passive transport, driven by the gradient in concentration and by the membrane potential. In fact, experiments demonstrated that in energy-depleted cells, where maintenance of membrane potential is defective, there was no Ca^2+^ influx (Eilam and Chernichovsky, 1987).

According to our simulations, even during steady-state conditions, and Ca^2+^ enters the cell through unstimulated MS channels. Indeed, most channels are known to be “leaky”, i.e., they stochastically open even in non signaling conditions. Since the flux through a single channel can be several orders of magnitude higher than that of a single transporter (Milo et al., 2010), even brief channel openings can theoretically supplement the cytosol with a significant amount of Ca^2+^. It has been shown that treatment with gadolinium, a blocker of stretch-activated channels, eliminates the Ca^2+^ rise in response to HTS without significantly affecting the steady-state Ca^2+^ concentration (Batiza et al., 1996). Accordingly, removing MS channels from our model decreases steady-state calcium levels from 215 to 160 nM, a value which is still well within the physiological range (Cunningham K. W. and Fink G. R., 1994). During model definition, a feedback inhibition was introduced on MS channels, mediated by calmodulin. This is the only assumption made in the model that is not yet supported by strong experimental evidence. We suggest that some kind of calcium-dependent feedback must exist, which either increases the activity of intracellular transporters or decreases the activity of MS channels. Many ion channels are inhibited by calmodulin (Saimi and Kung, 2002) and performing a parameter estimation with the model lacking this feedback inhibition produced poor results, in particular for the *cnb1*Δ mutant.

HACS channel has been shown to be activated following a number of external stimuli, such as alkaline stress and mating pheromone (Iida et al., 1994; Viladevall et al., 2004), but it is also involved in calcium uptake during normal growth, as evidenced by long-term calcium accumulation studies (Muller et al., 2001). In addition, HACS seems to physically interact with, and be inhibited by, calcineurin (Muller et al., 2001; Bonilla and Cunningham, 2003). After the parameter estimation process, it emerged that in our model the equation describing this negative regulation was unnecessary to reproduce the experimental data. This result suggests that, in the growth conditions considered here, feedback inhibition by calcineurin does not change significantly, since calcineurin activity is low in the chosen cultural conditions (Groppi et al., 2011).

Flc2 belongs to a TRP-like fungal family of putative yeast calcium transporters, together with Yor365c, Flc1, and Flc3 (Tisi et al., 2016). This raises the possibility that also the MS channels, with which Flc2 seems to interact, might belong to the aforementioned family. Our simulations support the hypothesis that Flc2 exerts an inhibitory role on the MS channels located on the plasma membrane (Rigamonti et al., 2015). In fact, the observed increase of calcium response and calcineurin hyperactivation in the *flc2*Δ mutant (Rigamonti et al., 2015) could be reproduced with the model just by increasing the activity of the MS channels. Flc2 may directly or indirectly influence the MS channel by affecting its stability, function or localization.

**FIGURE 7:**
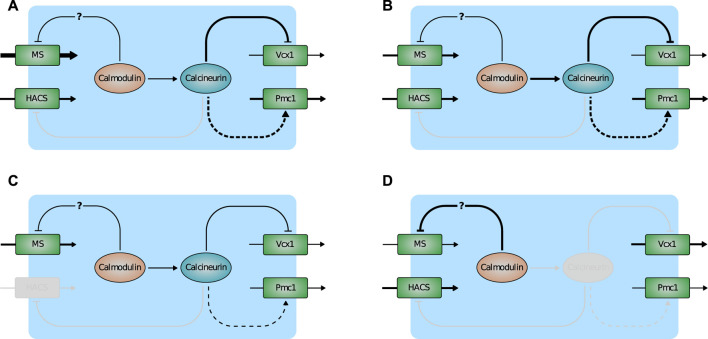
Scheme depicting the main predictions of the model. **(A)** Wild-type. **(B)** *flc2*Δ. **(C)** *cch1*Δ. **(D)** *cnb1*Δ. Activation is indicated by arrows, inactivation by T-bar arrows. Plain and dashed lines indicate post-transcriptional and transcriptional regulations. Line thickness represents the strenght of fluxes/(in)activations. Inactive components of the pathway are greyed-out.

Most of the model predictions are in accordance with experimental results that were not used for the parameter estimation process, suggesting that the model provides an accurate description of the HTS response in yeast cells as well as the most relevant transcriptional and post-transcriptional regulations involved in calcium handling. In particular, the model suggests the following regulatory scheme (Figure 7). Normally, Ca^2+^ enters the cell via HACS and MS channel leak. Pmc1 is the main intracellular transporter that keeps Ca^2+^ cytosolic level within the physiological range by pumping it into the vacuole. In the wild-type, Vcx1 is almost completely inhibited by calcineurin (Figure 7A). Mutants defective in HACS channel are still able to maintain physiological levels of cytosolic Ca^2+^ because calcineurin-dependent expression of Pmc1 is decreased. For the same reason, when this strain is challenged with HTS, the sudden Ca^2+^ influx is more slowly attenuated by Pmc1 (Figure 7C). In *flc2*Δ mutants, the flux through the MS channels is increased by removal of the inhibitory effect of Flc2 (Figure 7B). Lastly, mutants lacking calcineurin express less Pmc1, but the inhibitory effect on Vcx1 is relieved. Vcx1 only partially compensates for the reduced Pmc1 activity and the resulting higher Ca^2+^ levels activate calmodulin, that in turn inhibits MS channels (Figure 7D).

The function of the Ca^2+^ transient is still unclear. Calcineurin regulates gene expression by promoting Crz1 movement into the nucleus (Stathopoulos and Cyert, 1997; Cai et al., 2008). During HTS, Crz1 stays in the nucleus for about 5 mins (Oh et al., 2012), but its activity is only slightly increased (Rigamonti et al., 2015). In mammalian cells, calcium is often required for regulatory volume decrease (Jakab et al., 2002), while in yeast cells it could be implicated in the regulation of the cell wall integrity pathway (Levin, 2011; Rigamonti et al., 2015). In addition to the transient increase of Ca^2+^, HTS rapidly stimulates the Plc1-dependent hydrolysis of phosphatidylinositol 4,5-bisphosphate (Perera et al., 2004), an event that in many cell types elicits complex calcium responses (Hille et al., 2015). However, yeast *plc1*Δ mutants are still able to generate the Ca^2+^ signal during HTS (Tisi et al., 2002) and, conversely, Plc1 activity is independent from calcium availability in the medium (Perera et al., 2004), suggesting that in yeast these events might be independent.

## 7 Conclusion

As extensively described, the simulations of our model are in agreement with the experimental data, suggesting that the cellular components included in the model and their interactions are sufficient to explain the biological data in our hands. The model was defined on the basis of experimental evidences from the literature, and the parameter estimation process was performed against data from both the wild-type and mutant cells, in order to maximise the prediction’s reliability. The model presented in this work could be extended to include the response to several other stimuli known to elicit a calcium signal in yeast (Iida et al., 1990; Courchesne and Ozturk, 2003; Groppi et al., 2011). Such extensions of the model can be achieved either by modifying the current system of ODEs, or by adopting hybrid modeling approaches (Spolaor et al., 2019b; Nobile et al., 2020), in order to include other functional modules in the model that can better describe the different cellular processes involved in Ca^2+^ signalling (including, e.g., gene regulation and expression). Extended versions of the model could be useful for understanding the role of known channels and transporters, suggesting novel putative regulatory mechanisms and gaining new insights on Ca^2+^ signaling that can be further investigated experimentally.

Being Ca^2+^ an essential signaling ion, proteins involved in its homeostasis are increasingly studied as potential targets of antifungal drugs (Kwun et al., 2021; Li et al., 2021; Wang et al., 2021). A model of Ca^2+^ signaling could be used to predict the outcomes of the inhibition of a particular protein on the calcium physiology of fungal cells, thus helping to identify the best drug targets. Moreover, such models could be used to study *in silico* the relationships between pathogenic fungi and human cells (Spolaor et al., 2019a), accelerating the discovery of new antifungal treatments. Since Ca^2+^ signaling is well conserved among fungi (Tisi et al., 2016), a model of *S. cerevisiae* would require only slight modifications to describe what happens in other, pathogenic fungi.

Finally, as a future development, we plan to perform a large-scale sensitivity analysis of the model, in order to determine the most relevant parameters governing the response to hypotonic shock. Global sensitivity analysis requires a massive amount of independent simulations, which can lead to a huge computational effort (Nobile and Mauri, 2017). In order to make this analysis feasible, we will re-implement the model using the Python library ginSODA (Nobile et al., 2019), which provides the possibility of offloading a massive number of simulations of an ODE-based model to the GPU, thus strongly reducing the overall running time.

## Data Availability

The model presented in this study can be found in the BioModels repository, with accession number ID MODEL2112030001, at the following link https://www.ebi.ac.uk/biomodels/MODEL2112030001.

## Footnotes

1

$\bar{V}$ for glycerol is 0.071 ⋅ 10^−3^ *m* ^3^/*mol*
